# A Minor Surgical Operation for the Prevention of Conception, and Its Applications

**Published:** 1906-09

**Authors:** J. W. Kenney

**Affiliations:** San Antonio, Texas


For Texas Medical Journal.

### A Minor Surgical Operation for the Prevention of Conception, and its Applications.

BY J. W. KENNEY, M. D., SAN ANTONIO, TEXAS.

The desirability of preventing the fecundation of the ovum in certain conditions is self-evident. It is demanded in many instances. This desirability and demand has caused the manufacture of many and varied mechanical contrivances and chemical concoctions. Most of these are useless, many are injurious, and all a nuisance.

The attention given the subject by surgeons is unworthy of note. In fact, the literature on the subject is almost as scarce as the proverbial hen's teeth.

When the frequency with which we are consulted in regard to the prevention of conception is taken into consideration, it is seemingly strange that its importance has not been realized or at least given more consideration. The answers given to such consultants are usually intended to be humorous, yet, in reality, indicate a lack of knowledge—a lack of appreciation of the importance of the question asked.

The operation which I shall describe is never injurious, nor is it a nuisance, while, on the other hand, it has been very successful in my hands. It reduces the probability of conception to a minimum; in fact, has prevented it altogether, when satisfactorily performed, in my practice during the past four years.

The operation consists simply in the placing of a valve by surgical means in the cervical canal. To do this two incisions are made in the anterior cervical lip—one on either side of the mesial line and about one-eighth of an inch from it. The tongue thus made is depressed and the edges of the two incisions are brought together over it. Some trimming is necessary to make the operation an artistic one. This, however, will suggest itself to the operator. We thus form a valve-like projection into the cervical canal which acts as a practical barrier to anything entering the cervix, while, on the other hand, it but slightly obstructs the passage of anything from the womb.

A condition similar to the one artificially produced as just described is often found to exist naturally in the shape of small tumors in the cervix. The sterility of these women is frequently cured by the removal of these small growths. In like manner, the sterility artificially produced can be relieved at any time such a course may be deemed necessary, by the removal of the surgically-constructed valve in the cervical canal.

That it is the province of the medical profession, in so far as it is within their power, to legitimately regulate the quality of mankind born into the world does not admit of just criticism. Such supervision is exercised over all other animals and even plant life. Why not treat the human family like kindly? There are few among us who would not think of propagating a species of maimed, crippled or deformed or diseased horses, while there are many who are blindly propagating defectives in the human family. That such a state of affairs is wrong, none will deny. There are among us, however, those who will honestly object to any procedure that will interefere with what they are pleased to term "nature," on moral grounds. To such I can only say that the propogation of a race of idiots or humpbacks is a violation of the laws of God, and should be of man. To their argument regarding laws forbidding the marriage of such persons it can be stated that such laws are ineffective and for obvious reasons will ever be so. When instinct shows itself as passion, reason becomes subservient and laws will be of no avail. The fact that a genius sometimes makes his appearance among the vast throng of diseased and deformed is the only sane argument that can be advanced. Yet I doubt not if such an one were asked if life was worth living he would in truth unhesitatingly say "no." Should they for one minute stop to consider the humiliation and misery that is in store for their own offspring, I am sure their answer would be unhesitatingly and emphatically in the negative.

Chief among the conditions calling for some means of preventing conception may be mentioned consumption, mental degenerates, syphilis, deformities and maternal decay. The elimination of conception in parties suffering with the first named disease would relieve all orthopedic hospitals of more than half their charge, while in the last mentioned the prevention of conception would give health and happiness to many a mother whose life and strength is needed to properly rear those whom she has already caused to be on earth.

The subject of preventive medicine presents many alluring fields for study, yet none can exceed in importance the successful carrying out of the object for which the minor surgical procedure prompting this paper was originated. All good things meet with abuse, and should a simple procedure for the prevention of conception be placed in that class, it will likewise be abused. The desire for the operation becomes more and more apparent as its simplicity and reality become more generally known. There will be those who will ask its performance as a matter of convenience —simply to relieve them of the burdens of maternity. There will be others who will seek its performance that they may illicitly unbridle their passions without fear of their sin leading to public shame. The performance of this operation, or a better one for the same purpose, will always be in the hands of the medical profession, and I feel that I can say of the members of my profession, as Longfellow did of women, "Even doctors in their deepest degradation hold something sacred; something undefiled; some precious keepsake of their higher and better nature, and, like a diamond in the dark, still retain some quenchless gleams of the celestial fire."

